# Forest Density and Invasive Carnivores Are Related to *Trichinella* Infection in Wild Boars in Poland

**DOI:** 10.3390/pathogens14090906

**Published:** 2025-09-09

**Authors:** Jakub Kubacki, Daniel Klich, Aneta Bełcik, Weronika Korpysa-Dzirba, Tomasz Cencek, Jacek Karamon, Jacek Sroka, Małgorzata Samorek-Pieróg, Michał Gondek, Ewa Bilska-Zając

**Affiliations:** 1 General Veterinary Inspectorate, Wspólna Street 30, 00-930 Warsaw, Poland; 2 Department of Animal Genetics and Conservation, Institute of Animal Sciences, Warsaw University of Life Sciences (SGGW), Nowoursynowska Street 166, 02-787 Warsaw, Poland; daniel_klich@sggw.edu.pl; 3 Department of Parasitology and Invasive Diseases, Bee Diseases and Aquatic Animal Diseases, National Veterinary Research Institute, Partyzantów Avenue 57, 24-100 Puławy, Poland; weronika.korpysa@piwet.pulawy.pl (W.K.-D.); tcencek@piwet.pulawy.pl (T.C.); j.karamon@piwet.pulawy.pl (J.K.); jacek.sroka@piwet.pulawy.pl (J.S.); malgorzata.samorek-pierog@piwet.pulawy.pl (M.S.-P.); ewa.bilska@piwet.pulawy.pl (E.B.-Z.); 4 Department of Food Hygiene of Animal Origin, Faculty of Veterinary Medicine, University of Life Sciences in Lublin, Akademicka 12, 20-033 Lublin, Poland; michal.gondek@up.lublin.pl

**Keywords:** *Trichinella* spp., trichinellosis, zoonosis, wild boar, raccoon dog, density

## Abstract

The purpose of this study was to investigate and update the spatial distribution of *Trichinella* spp. in wild boars tested between 2015 and 2022 and to test the correlation of the population density of chosen animals (wild boars, red foxes (*Vulpes vulpes*), raccoon dogs (*Nyctereutes procyonoides*), and European badgers (*Meles meles*)) with the prevalence of *Trichinella* spp. in wild boars in Poland. In addition, to understand the distribution of infected animals, we sought to see if there were a correlation of *Trichinella* spp. infections in wild boars with land cover type. Among the wild carnivore species analyzed, only the population density of the raccoon dog (*Nyctereutes procyonoides*)—an invasive alien species—was significantly associated with infection rates in wild boars, particularly at the regional scale. As scavengers and competent reservoir hosts for all four European *Trichinella* species, raccoon dogs are likely to play a key role in the sylvatic transmission cycle. The positive rate of *Trichinella* spp. infection in wild boars during 2015–2022 was 0.22%, compared to 0.3% in 2009–2016. Moreover, forest density was positively correlated with infection rates, underlining the role of forest habitats in sustaining *Trichinella* transmission.

## 1. Introduction

*Trichinella* spp. are parasitic nematodes that cause trichinellosis, a zoonotic disease transmitted primarily through the consumption of meat from infected animals containing live larvae [1]. The global distribution of *Trichinella*, along with its ability to infect a wide range of hosts, including mammals, birds, and reptiles, makes it an important pathogen of both veterinary and public health significance [2,3]. While domestic animals, especially pigs (*Sus domestica*), have historically been the primary reservoirs for human infection, increasing evidence suggests that wild animal populations play a crucial role in the epidemiology of *Trichinella* spp. in Europe [4,5]. Wild boars (*Sus scrofa*) are considered the most significant source of *Trichinella* infection in humans in the majority of European countries, particularly in regions where hunting for wild game meat is common [6,7]. In Europe, where wild boar populations are widespread and often coexist with humans in rural and peri-urban areas and the meat of wild boars is growing in popularity, the risk of zoonotic transmission through ingestion of hunted animals has become a growing concern [2,7,8]. Therefore, monitoring and understanding the prevalence of *Trichinella* in wild boar populations has become increasingly important in controlling the spread of the disease [9,10,11].

As is well known, the epidemiology of *Trichinella* in wild animals varies greatly across countries and is influenced by a variety of factors, including host species, geographical location, ecological and environmental circumstances, and the character of wildlife–human interactions [4,8,12,13,14].

The major hosts with significant implications in the distribution of these parasites in sylvatic environments are carnivores and omnivores [15,16,17,18]. These animals can serve as both reservoirs and vectors, maintaining the parasite in the environment and facilitating its transmission to other species, including domestic animals [18]. Their importance in the distribution of the pathogen depends especially on the number and density of the host population. The epidemiological importance of particular carnivore species, however, varies geographically. While some studies suggest that mesocarnivores such as raccoon dogs may represent efficient reservoir hosts, the role of red foxes and badgers appears more limited in certain regions—for example, López-Olvera et al. [19] found a very low prevalence in foxes in Catalonia, Spain. Thus, the contribution of these native carnivores to the sylvatic cycle in Poland remains uncertain and warrants cautious interpretation.

The results of our previous study, which reported the status for the 2009–2016 period, show that the positive rate of *Trichinella* spp. in wild boars in Poland amounted to 0.3%. in the northwestern regions of our country, the number of wild boars infected with *Trichinella* spp. was significantly higher than in southern provinces [15]. Similar observations were noted concerning the geographical distribution of infected red foxes [15]. Some exceptions in the case of wild boar populations occurred, e.g., in a central province, namely, Świętokrzyskie, where, in some counties, the number of infected wild boars was at a similarly high level to that in northwestern counties [15].

The purpose of this study was to investigate and update the spatial distribution of *Trichinella* spp.-infected wild boars tested between 2015 and 2022 and to test the correlation of the population density of chosen animals (wild boars, red foxes (*Vulpes vulpes*), raccoon dogs (*Nyctereutes procyonoides*), and European badgers (*Meles meles*)) with the prevalence of *Trichinella* spp. in wild boars in Poland. In addition, to understand the distribution of infected animals, we sought to see if there was a correlation of *Trichinella* spp. infections in wild boars with land cover type.

## 2. Material and Methods

This study was based on counties, the smallest administrative units for which data on wild boars’ infection with *Trichinella* are collected in Poland by the General Veterinary Inspectorate (GVI). For this study, we obtained four types of data: (a) number of infected wild boars with *Trichinella* in each county (Polish administrative unit—county) for six hunting seasons: 2015/16–2021/22 (data obtained from GVI), (b) number of hunted wild boars and number of carnivores (red fox, raccoon dog, and European badger) for six hunting seasons: 2015/16–2010/22 (data obtained from Forest Data Bank in Poland; https://www.bdl.lasy.gov.pl/portal/) [20], (c) land cover for the year 2018 (data obtained from Corine Land Cover (CLC); https://clc.gios.gov.pl/) [21], and (d) vector layers of administrative units of Poland (data obtained from official website Open Data; https://dane.gov.pl/pl) [22].

Prior to the analysis, the data was prepared to create appropriate variables. The number of infected and hunted wild boars during hunting seasons 2015/16–2021/22 were summed, and then the number of infected wild boars per 10,000 hunted wild boars in each county was calculated. Basing on Corine Land Cover (CLC), we selected four main cover types: settlements (SETT; CLC classes: 1.1.1 and 1.1.2), crops (CROPS; mainly arable land and permanent crops; all CLC classes: 2), forests and seminatural vegetation (FOREST; areas covered with natural woody vegetation, including forests and shrub vegetation; CLC classes: 3.1 and 3.2), and water bodies (WATER; CLC classes: 4.1.1, 4.1.2, 5.1.2). Using vector layers of counties’ boundaries, we calculated the percentage of each of these four cover types in each county. Based on the numbers of three mesocarnivore species and wild boars, we calculated the average density of these species in each county (number of animals per 1000 ha).

We excluded from the analysis all cities with county rights due to the fact that game animals are usually managed there with greater restrictions and their numbers are more difficult to assess because they are excluded from yearly hunting plans

**Figure 1:**
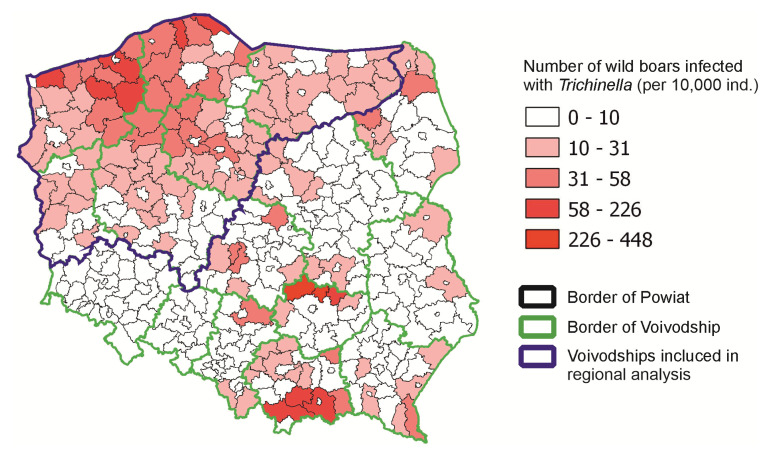
Number of wild boars infected with *Trichinella* during the hunting seasons 2015/16–2021/22 in Poland.

We performed the analysis at two levels: the national and regional levels. Our approach resulted from the fact that infections of wild boars with *Trichinella* in Poland varied significantly ([15] and Figure 1). In a large part of the country, few infections of wild boars were detected, but there were also areas where such infections were numerous. For this reason, in addition to the analysis at the national level, we added an analysis at the regional level (the northwestern part of the country) to check whether the demonstrated trends were confirmed in an area with a high level of wild boar infection (Figure 1). Finally, we used the following numbers of counties in the analysis: *n* = 310 for the country level and *n* = 115 for the regional level. The number of infected wild boars per 10.000 hunted wild boars in each county was applied to vector layers in QGIS to present the geographical distribution of infected wild boars in Poland.

To evaluate the impact of environmental factors on wild boars’ infection with *Trichinella*, we used a generalized linear model with a negative binomial distribution and a log link function. We applied a generalized linear model because the response variable was not normally distributed and transformations failed. Negative binomial models can cope with overdispersion in the count data [23]. In the model, the dependent variable was the number of infected wild boars per 10,000 hunted wild boars. We assumed eight explanatory variables: density of three mesocarnivores (red fox (FOX), raccoon dog (RACC), and European badger (BADG)); density of wild boars (BOAR); and the percentage of four cover types (settlements (SETT), forests (FOREST), crops (CROPS), and water bodies (WATER). We also checked the correlation of explanatory variables, i.e., land cover types and mesocarnivore density using Pearson’s correlation coefficient (we used r = |0.7| as the collinearity threshold). As the correlation between FOREST and CROPS was very high (r = 0.919 for the country level, and r = 0.917 for the regional level), we excluded crops from further analysis. Finally, we analyzed the impact of seven environmental variables on wild boars’ infection with *Trichinella*. Model selection was performed similarly for both approaches, i.e., country and regional level (*n* = 310 for country level and *n* = 115 for regional level). Namely, we performed all model variants, including the null model, to find the highest ranked model based on Burnham and Anderson (2002). All preformed models were ranked according to the Akaike Information Criterion (AIC) value. The model with the lowest AIC was regarded as the most fitting and highest ranked. Results of the highest ranked models are presented. All statistics were analyzed with IBM SPSS Statistics 29.0 (Armonk, NY, USA).

## 3. Results

Between 2015 and 2022, data of 4275 infected wild boars in Poland were reported by GVI. There were no provinces where *Trichinella* were not found. The highest number of infected wild boars was reported for the northwestern provinces, with large differences between counties. The analysis of the number of infected wild boars per 10,000 hunted wild boars in each county between 2015 and 2022 is presented on a map (Figure 1). For the analysis at the country level, 310 counties were used, while for regional analysis, six provinces with 115 counties were applied. The details are presented in Table 1.

**Figure 2:**
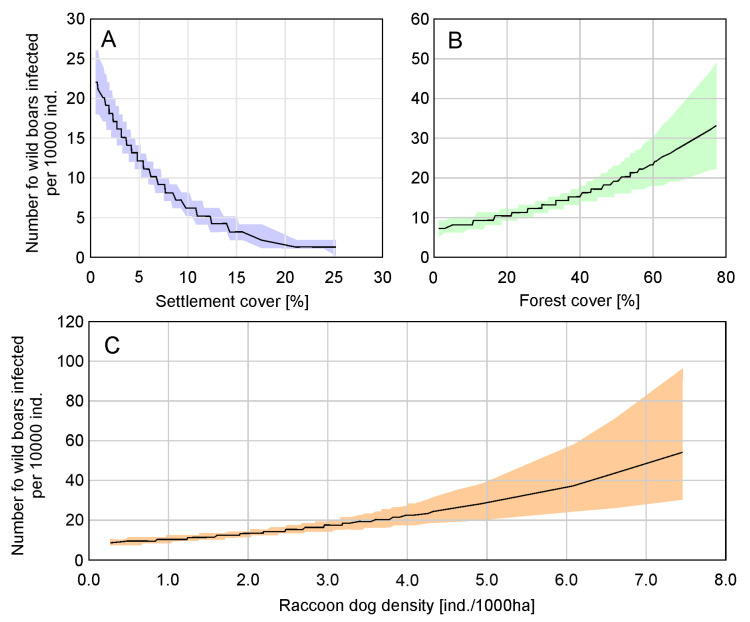
Relation of wild boars’ infection with *Trichinella* and (**A**) area covered by settlements, (**B**) area covered by forests, and (**C**) raccoon dog density based on country level analysis with generalized linear model.

The highest-ranking model in the country level analysis included four variables: RACC, SETT, FOREST, and WATER (Table 2), and other variables were excluded during model selection (Supplementary Materials Table S1). It is worth noting that within ΔAIC = 2, there were six models, where the variables FOX, BADG, and BOAR were also present. In these models, these variables were non-significant. The highest ranked model presented a low ΔAIC (0.6), with the second ranked model containing only RACC, SETT, and FOREST, but there was a high ΔAIC with the null model (68.7), which was ranked as 127 (Table S1). Wild boars’ infection with *Trichinella* significantly depended on the settlement (*p* = 0.003) and forest percentage (*p* < 0.001) (Table 2), but the relation differed. The number of wild boars infected increased with the share of forest (FOREST) but decreased with the share of settlements (SETT) (Table 2, Figure 2). Moreover, the number of wild boars infected increased with the raccoon dog density (RACC) (*p* = 0.020) (Table 2, Figure 2). Water bodies (WATER), although present in the highest ranked model, did not significantly impact wild boars’ infection with *Trichinella* (*p* = 0.127) (Table 2).

**Figure 3:**
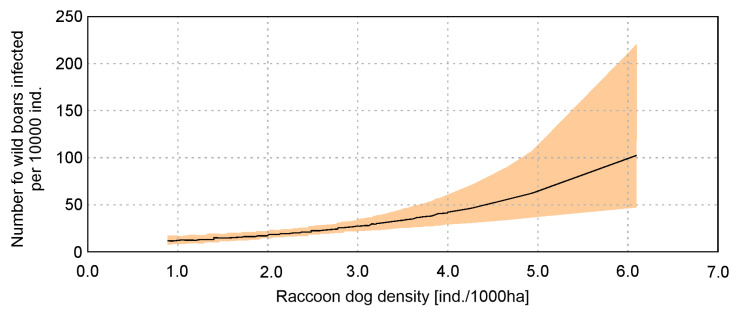
Relation of wild boars’ infection with *Trichinella* and raccoon dog density based on regional level analysis with generalized linear model.

The highest ranked model in the regional level analysis included only raccoon dog density (RACC); all other variables were excluded during model selection (Supplementary Materials Table S1). However, there are a large number of models within ΔAIC = 2 (12 models), and Akaike weights of the highest ranked model were low (ωi = 0.69). Other models within ΔAIC = 2 contained all variables used in the analysis (Table S1), but the ΔAIC between the highest ranked model and the null model equaled 16.8. Raccoon dog density significantly influenced the infection of wild boars with *Trichinella* (*p* < 0.001) (Table 3). This relation was stronger than in country-level analysis, but there were also larger Confidence Intervals for higher values of raccoon dog density (Figure 3).

## 4. Discussion

The prevalence of trichinellosis in wild boars found in this study (2015–2022, 0.22%) compared to the previous study period (2009–2016, 0.3%) suggests a slight decrease, highlighting temporal changes in the dynamics of *Trichinella* infections in wild boars. In comparison, the prevalence of *Trichinella* infections in humans in Poland during 2015–2022 totaled 67 reported cases (an average of 8.4 cases per year), with the highest burden recorded in 2015 (27 cases) and a marked decline thereafter. These data indicate that although infections in wild boars continue to occur and are geographically dispersed, the risk of transmission to humans is relatively low and has been decreasing in recent years. The analysis of the number of infected wild boars in each county between 2015 and 2022 confirms previous findings for the spatial distribution of *Trichinella*-infected wild boars [15] (Figure 1). Based on the data obtained, we distinguished three geographic regions that are endemic areas of trichinellosis in wild boars. One of these regions—northwestern Poland—is extensive and covers dozens of counties, another one located in Świętokrzyskie province covers only three counties and their surroundings, and the last region located in Małopolskie province also covers three counties and their surroundings. The area of northwestern Poland is rich in forests, especially in the three provinces located furthest to the north and northwest, where forest cover reaches nearly 40%. This environment provides favorable conditions for settlements of wild boars. The Świętokrzyskie and Małopolskie provinces have slightly less forested lands (28.5% and 28.6%, respectively), and the amount of forested areas in the two provinces mentioned above does not differ significantly from the percentage of forested land in most other parts of Poland [15]. Wild boars prefer dense forest habitats, especially those formed by tree species that provide high-energy food, such as oak acorns and beech nuts [22]. Therefore the results of statistical analysis at the country level, indicating that the higher the percentage of the county’s area of forests and the lower the percentage of settlements, the higher the number of wild boars infected with *Trichinella* spp., are not surprising, yet underline the importance of habitat in wild boar populations, which is strictly linked with forests in which a richness of food is found for these animals. We did not find a correlation between the area of water bodies and *Trichinella* infections in wild boars. Although water environments can indirectly influence parasite transmission through host distribution and behavior, current results do not support a significant role. Some previous reports (e.g., in northern Europe) have suggested potential ecological links between aquatic habitats and carnivore feeding behavior, but such associations were not observed here. We identified a significant positive correlation of raccoon dog density with the number of infected wild boars, whereas no significant correlation was found for red fox, badger, or wild boar population density. This finding supports the hypothesis that raccoon dogs, as invasive and highly adaptable scavengers, represent particularly important hosts for maintaining the sylvatic cycle of *Trichinella* in Poland. Although red foxes and badgers were included in our analysis, their densities did not significantly affect wild boar infection rates. This result is in line with studies from other European regions, which often report a low prevalence of *Trichinella* in these species (e.g., López-Olvera et al. [19]). Therefore, while these native mesocarnivores may occasionally contribute to transmission, their epidemiological role in Poland appears secondary compared to that of the raccoon dog. Future studies using direct larval detection and molecular confirmation (PCR) are needed to better assess their real contribution.

Raccoon dogs are mesocarnivores originating from East Asia, and in Europe, they are considered an invasive alien species [24]. The population was noticed for the first time in Poland in 1955 in eastern border provinces, and over decades, it has grown to a size that now covers an entire country [25]. These animals have a variable diet depending on the availability of different foodstuffs in given areas and seasons [26]. The most frequent diet during winter is carrion, which may constitute up to 76% of biomass consumed by raccoon dogs [27]. This habit plays a crucial role, considering *Trichinella* circulation in sylvatic environments. As a matter of fact, raccoon dogs are considered to be an indicator species and well-adapted reservoir hosts for all four *Trichinella* species present in Europe [16]. Going even further, in Finland, the raccoon dog not only serve as the sole host for all four *Trichinella* species but also exhibits the highest intensity of infections [17].

The occurrence of *Trichinella* spp. (*T. spiralis* and *T. britovi*) in raccoon dogs in Poland has been documented in only a few studies [28,29]. The latest study by Cybulska et al. [29] indicates a very important role of raccoon dogs in the distribution of *T. britovi* in the eastern part of Poland, where almost 40% of tested raccoon dogs were infected with this species. A similar number of raccoon dogs infected with *T. britovi* was found in Estonia, while in Lithuania, it was over 70% [11]. The most commonly identified *Trichinella* species in raccoon dogs in Finland was *T. nativa*, followed by *T. spiralis* and *T. britovi*, although Oksanen et al. [12] reported *T. nativa* as the predominant species followed by *T. britovi* [12,30]. Taking into account the data from Germany, which borders west Poland, *T. spiralis* dominates among raccoon dogs [31]. The data presented above indicate that the distribution of *Trichinella* spp. may be related to the environment and the climate [29].

In fact, in Poland, the majority of infected wild boars are infected with *T. spiralis*, particularly in the northwestern part of the country [32]. This distribution of *Trichinella* species (*T. spiralis* across the entire country and *T. britovi* mostly at the eastern border of the country and occasionally in the western part) indicates that the biomass of *T. spiralis* is very rich, especially in the western part of Poland [32]. The statistical analysis conducted in our study on a regional level, which took into account only the areas with high numbers of infected wild boars, shows that it may be correlated with raccoon dog population density. The significance of this correlation is statistically higher than in cases of statistical analysis calculated on the country level. In fact, in the study by Osten-Sacken and Solarczyk [28], 39 raccoon dogs accidentally killed on roads in Warta Mouth National Park were tested, and in 2 of them, *T. spiralis* was identified (5%). Moreover, a study of raccoon dogs conducted in the Brandenburg region in Germany neighboring northwestern Poland indicated that in this area, 4.8% of raccoon dogs were infected with *Trichinella*, and the majority (70–90%) were infected with *T. spiralis* [31]. The newest research by Johne et al. [33] confirms previous results in Germany and presents 27 raccoon dogs who tested positive for *Trichinella* (75% of them was *T. spiralis*), all of them localized at the eastern border of Germany.

In conclusion, the results of the statistical analysis conducted in our research are in line with the above studies and conjecture regarding the relevance of raccoon dogs in the spread of trichinellosis. As a rapidly expanding invasive species, raccoon dogs can significantly contribute to the spread of *Trichinella* nematodes through the sylvatic cycle in Europe. This observation should however be investigated empirically, which will be the goal of the next study.

## 5. Conclusions

This study demonstrates that *Trichinella* infections in wild boars in Poland are spatially clustered, with endemic foci in forest-rich northwestern provinces. Forest cover and raccoon dog density were identified as significant positive determinants, underlining the ecological role of this invasive carnivore and forest habitats in maintaining parasite transmission. These results have important implications for wildlife disease monitoring and zoonotic risk management.

## Figures and Tables

**Table 1:** Summary of *Trichinella* invasions in wild boars in Poland reported by GVI between 2015 and 2022.

Province	Number of Wild Boars Infected with *Trichinella* spp.	Prevalence	95% Confidence Intervals
dolnośląskie	65	0.03	0.02–0.03
kujawsko-pomorskie	347	0.33	0.30–0.37
lubelskie	62	0.08	0.06–0.10
lubuskie	208	0.14	0.12–0.15
łódzkie	81	0.12	0.10–0.15
małopolskie	192	0.29	0.25–0.33
mazowieckie	70	0.06	0.05–0.08
opolskie	52	0.06	0.05–0.08
podkarpackie	56	0.08	0.06–0.10
podlaskie	72	0.16	0.13–0.20
pomorskie	652	0.46	0.43–0.50
śląskie	63	0.07	0.06–0.09
świętokrzyskie	390	1.12	1.01–0.23
warmińsko-mazurskie	270	0.16	0.14–0.18
wielkopolskie	379	0.17	0.16–0.19
zachodniopomorskie	1316	0.40	0.38–0.43
All	4275	0.22	0.21–0.22

**Table 2:** Effect of raccoon dog density (RACC), area of settlements (SETT), area of forests (FOREST), and area of water bodies (WATER) on wild boars’ infection with *Trichinella* in the highest ranked generalized linear model on the country level (red fox and European badger and wild boar density were excluded during model selection; chi2 = 76.68, *p* < 0.001 for the highest ranked model).

Source	Beta	SE	χ^2^	*p*	Lower CI	Upper CI
Intercept	2.257	0.212	112.97	<0.001	1.841	2.673
RACC	0.138	0.060	5.38	0.020	0.021	0.255
SETT	−0.113	0.018	38.25	<0.001	−0.148	−0.077
FOREST	0.013	0.005	8.83	0.003	0.005	0.022
WATER	0.056	0. 036	2.38	0.127	−0.015	0.127

**Table 3:** Effect of raccoon dog density (RACC) on wild boars’ infection with *Trichinella* in the highest ranked generalized linear model on the regional level (other variables were excluded during model selection; chi2 = 18.82, *p* < 0.001 for the highest ranked model).

Source	Beta	SE	χ^2^	*p*	Lower CI	Upper CI
Intercept	1.992	0.272	53.55	<0.001	1.458	2.525
RACC	0.433	0. 105	17.14	<0.001	0.228	0.638

## Data Availability

The original contributions presented in the study are included in the article. Further inquiries can be directed to the corresponding authors.

## Supplementary Materials

The following supporting information can be downloaded at https://www.mdpi.com/article/10.3390/pathogens14090906/s1. Table S1: Ranking of the models (within *Ʃω_i_* = 0.95 and null model) explaining the number of wild boars infected with *Trichinella* in generalized linear models with negative binomial distribution and log link function (Δ*AIC*—*AIC* differences, *ω_i_*—Akaike weights, Rank—rank of the models based on *AIC* values; bolded text in the row indicates chosen model (variables: FOX—red fox density, BADG—European badger density, RACC—Raccoon dog density, BOAR—wild boar density, SETT—area of settlements, FOREST—area of forests, WATER—area of water bodies; for details: see methods).

## References

[1] Bruschi F. (2021). Trichinella and Trichinellosis.

[2] Pozio E. (2019). *Trichinella* and trichinellosis in Europe. Vet. Glas..

[3] Murrell K.D., Pozio E. (2011). Worldwide occurrence and impact of human trichinellosis, 1986–2009. Emerg. Infect. Dis..

[4] Pozio E. (2005). The broad spectrum of *Trichinella* hosts: From cold-to warm-blooded animals. Vet. Parasitol..

[5] Rostami A., Gamble H.R., Dupouy-Camet J., Khazan H., Bruschi F. (2017). Meat sources of infection for outbreaks of human trichinellosis. Food Microbiol..

[6] Rostami A., Riahi S.M., Ghadimi R., Hanifehpour H., Hamidi F., Khazan H., Gamble H.R. (2018). A systematic review and meta-analysis on the global seroprevalence of *Trichinella* infection among wild boars. Food Control.

[7] Pozio E. (2007). World distribution of *Trichinella* spp. infections in animals and humans. Vet. Parasitol..

[8] Pozio E. (1998). Trichinellosis in the European Union: Epidemiology, ecology and economic impact. Parasitol. Today.

[9] Bilska-Zając E., Różycki M., Chmurzyńska E., Marucci G., Cencek T., Karamon J., Bocian Ł. (2013). *Trichinella* species circulating in wild boar (*Sus scrofa*) populations in Poland. Int. J. Parasitol. Parasites Wildl..

[10] Kozar Z., Ramisz A., Kozar M. (1965). Incidence of *Trichinella spiralis* in some domestic and wild living animals in Poland. Wiad. Parazytol..

[11] Malakauskas A., Paulauskas V., Järvis T., Keidans P., Eddi C., Kapel C.M.O. (2007). Molecular epidemiology of *Trichinella* spp. in three Baltic countries: Lithuania, Latvia, and Estonia. Parasitol. Res..

[12] Oksanen A., Interisano M., Isomursu M., Heikkinen P., Tonanzi D., Oivanen L., Pozio E. (2018). *Trichinella spiralis* prevalence among wildlife of a boreal region rapidly reduced in the absence of spillover from the domestic cycle. Vet. Parasitol..

[13] Pozio E. (2022). The impact of globalization and climate change on *Trichinella* spp. epidemiology. Food Waterborne Parasitol..

[14] Pannwitz G., Mayer-Scholl A., Balicka-Ramisz A., Nöckler K. (2010). Increased prevalence of *Trichinella* spp., northeastern Germany, 2008. Emerg. Infect. Dis..

[15] Bilska-Zając E., Różycki M., Grądziel-Krukowska K., Bełcik A., Mizak I., Karamon J., Sroka J., Zdybel J., Cencek T. (2020). Diversity of *Trichinella* species in relation to the host species and geographical location. Vet. Parasitol..

[16] Kärssin A., Häkkinen L., Niin E., Peik K., Vilem A., Jokelainen P., Lassen B. (2017). *Trichinella* spp. biomass has increased in raccoon dogs (*Nyctereutes procyonoides*) and red foxes (*Vulpes vulpes*) in Estonia. Parasites Vectors.

[17] Oivanen L., Kapel C.M.O., Pozio E., La Rosa G., Mikkonen T., Sukura A. (2002). Associations between *Trichinella* species and host species in Finland. J. Parasitol..

[18] Bilska-Zając E., Różycki M., Korpysa-Dzirba W., Bełcik A., Ziętek-Barszcz A., Włodarczyk-Ramus M., Gontarczyk A., Cencek T. (2021). *Trichinella* outbreaks on pig farms in Poland in 2012–2020. Pathogens.

[19] López-Olvera J.-R., Vives L., Serrano E., Fernández-Sirera L., Picart L., Rossi L., Marco I., Bigas E., Lavín S. (2011). *Trichinella* sp. in red foxes (*Vulpes vulpes*) from Catalonia, NE Spain. Parasitol. Res..

[20] Forest Data Bank in Poland. https://www.bdl.lasy.gov.pl/portal/.

[21] Corine Land Cover (CLC). https://clc.gios.gov.pl/.

[22] Head Office of Geodesy and Cartography (GUGiK) Vector Layers of Administrative Units of Poland (National Register of Boundaries). https://dane.gov.pl/pl.

[23] Zuur A.F., Ieno E.N., Walker N.J., Saveliev A.A., Smith G.M. (2009). Mixed Effects Models and Extensions in Ecology with R.

[24] Mulder J.L. (2013). The raccoon dog (*Nyctereutes procyonoides*) in the Netherlands-its present status and a risk assessment. Lutra.

[25] Skorupski J. (2016). Inwazyjne i ekspansywne ssaki drapieżne w Polsce. Stud. Mater. Cent. Edukac. Przyr.-Leśn..

[26] Sutor A., Schwarz S., Conraths F.J. (2014). The biological potential of the raccoon dog (*Nyctereutes procyonoides*, Gray 1834) as an invasive species in Europe—New risks for disease spread?. Acta Theriol..

[27] Sidorovich V.E., Polozov A.G., Lauzhel G.O., Krasko D.A. (2000). Dietary overlap among generalist carnivores in relation to the impact of the introduced raccoon dog *Nyctereutes procyonoides* on native predators in northern Belarus. Z. Saugetierkd..

[28] Osten-Sacken N., Solarczyk P. (2016). *Trichinella spiralis* in road-killed raccoon dogs (*Nyctereutes procyonoides*) in western Poland. Ann. Parasitol..

[29] Cybulska A., Kornacka A., Moskwa B. (2019). The occurrence and muscle distribution of *Trichinella britovi* in raccoon dogs (*Nyctereutes procyonoides*) in wildlife in the Głęboki Bród Forest District, Poland. Int. J. Parasitol. Parasites Wildl..

[30] Airas N., Saari S., Mikkonen T., Virtala A.-M., Pellikka J., Oksanen A., Isomursu M., Kilpelä S.-S., Lim C.W., Sukura A. (2010). Sylvatic *Trichinella* spp. infection in Finland. J. Parasitol..

[31] Mayer-Scholl A., Reckinger S., Schulze C., Nöckler K. (2016). Study on the occurrence of *Trichinella* spp. in raccoon dogs in Brandenburg, Germany. Vet. Parasitol..

[32] Bilska-Zając E., Franssen F., Różycki M., Swart A., Karamon J., Sroka J., Zdybel J., Ziętek–Barszcz A., Cencek T. (2019). Intraspecific genetic variation in *Trichinella spiralis* and *Trichinella britovi* populations circulating in different geographical regions of Poland. Int. J. Parasitol. Parasites Wildl..

[33] Johne A., Sachsenröder J., Richter M., Nöckler K. (2025). *Trichinella* findings in Germany from 2013 to 2023 indicate an increased prevalence in wild boar (*Sus scrofa*) population. Vet. Parasitol..

