# Supplementary material for: Forest Density and Invasive Carnivores Are Related to Trichinella Infection in Wild Boars in Poland
Source: Pathogens. 2025 Sep 9;14(9):906. doi: 10.3390/pathogens14090906 (PMC12472544; doi:10.3390/pathogens14090906)
Supplement: Supplementary file 1 [file pathogens-14-00906-s001.zip › pathogens-3793967-supplementary.pdf]

Supplementary Materials

Table S1. Ranking of the models (within $\Sigma\omega_i = 0.95$ and null model) explaining the number of wild boars infected with *Trichinella* in generalized linear models with negative binomial distribution and log link function (*ΔAIC - AIC* differences, ω_i - Akaike weights, Rank - rank of the models based on *AIC* values; bolded text in the row indicates chosen model (variables: FOX – red fox density, BADG - European badger density, RACC – Raccoon dog density, BOAR – wild boar density, SETT – area of settlements, FOREST – area of forests, WATER – area of water bodies; for details: see methods).

Models	***ΔAICc***	ω_i	***Rank***
Country Level			
**RACC + SETT + FOREST + WATER**	**0.0**	**0.169**	**1**
RACC + SETT + FOREST	0.6	0.125	2
RACC + SETT + FOREST + WATER + BOAR	1.4	0.084	3
RACC + SETT + FOREST + BOAR	1.9	0.065	4
FOX + RACC + SETT + FOREST + WATER	2.0	0.062	5
BADG + RACC + SETT + FOREST + WATER	2.0	0.062	6
FOX + RACC + SETT + FOREST	2.5	0.048	7
BADG + RACC + SETT + FOREST	2.5	0.048	8
BADG + RACC + SETT + FOREST + WATER + BOAR	3.3	0.032	9
FOX + RACC + SETT + FOREST + WATER + BOAR	3.4	0.031	10
SETT + FOREST + WATER	3.5	0.029	11
FOX + RACC + SETT + FOREST + BOAR	3.8	0.025	12
BADG + RACC + SETT + FOREST + BOAR	3.8	0.025	13
FOX + BADG + RACC + SETT + FOREST + WATER	3.9	0.024	14
FOX + SETT + FOREST + WATER	4.2	0.021	15
FOX + BADG + RACC + SETT + FOREST	4.5	0.018	16
BADG + SETT + FOREST + WATER	4.5	0.018	17
SETT + FOREST + WATER + BOAR	5.0	0.014	18
FOX + BADG + RACC + SETT + FOREST + WATER + BOAR	5.3	0.012	19
FOX + BADG + RACC + SETT + FOREST + BOAR	5.8	0.009	20
FOX + BADG + SETT + FOREST + WATER	5.9	0.009	21
FOX + SETT + FOREST + WATER + BOAR	5.9	0.009	22
BADG + SETT + FOREST + WATER + BOAR	6.3	0.007	23
RACC + SETT + WATER	6.9	0.005	24
…			…
null model	68.7	0.000	127
Regional Level			
**RACC**	**0.0**	**0.069**	**1**
RACC + SETT	0.1	0.066	2
RACC + SETT + BOAR	0.4	0.057	3
RACC + BOAR	1.0	0.042	4
BADG + RACC + SETT	1.3	0.036	5
BADG + RACC	1.3	0.036	6
FOX + RACC	1.4	0.035	7
RACC + FOREST	1.6	0.031	8
BADG + RACC + SETT + BOAR	1.9	0.027	9
FOX + BADG + SETT	1.9	0.027	10
RACC + WATER	1.9	0.027	11
RACC + SETT + FOREST	2.0	0.026	12
RACC + SETT + WATER	2.1	0.024	13
RACC + SETT + FOREST + BOAR	2.3	0.022	14
FOX + RACC + SETT + BOAR	2.3	0.022	15
RACC + SETT + WATER + BOAR	2.4	0.021	16
FOX + RACC + BOAR	2.5	0.020	17
BADG + RACC + BOAR	2.6	0.019	18
BADG + RACC + FOREST	2.8	0.017	19

FOX + BADG + RACC	2.9	0.016	20
RACC + WATER + BOAR	2.9	0.016	21
RACC + FOREST + BOAR	2.9	0.016	22
BADG + RACC + SETT + FOREST	3.1	0.015	23
FOX + BADG + RACC + SETT	3.3	0.013	24
BADG + RACC + SETT + WATER	3.3	0.013	25
BADG + RACC + WATER	3.3	0.013	26
FOX + RACC + FOREST	3.3	0.013	27
FOX + RACC + WATER	3.4	0.013	28
RACC + FOREST + WATER	3.6	0.012	29
FOX + BADG + RACC + SETT + BOAR	3.9	0.010	30
FOX + RACC + SETT + FOREST	3.9	0.010	31
BADG + RACC + SETT + FOREST + BOAR	3.9	0.010	32
FOX + RACC + SETT + WATER	3.9	0.010	33
BADG + RACC + SETT + WATER + BOAR	3.9	0.010	34
RACC + SETT + FOREST + WATER	4.0	0.009	35
FOX + RACC + SETT + FOREST + BOAR	4.2	0.009	36
FOX + BADG + RACC + BOAR	4.2	0.009	37
FOX + RACC + SETT + WATER + BOAR	4.3	0.008	38
RACC + SETT + FOREST + WATER + BOAR	4.3	0.008	39
BADG + RACC + FOREST + BOAR	4.4	0.008	40
BADG + RACC + WATER + BOAR	4.5	0.007	41
FOX + RACC + WATER + BOAR	4.5	0.007	42
FOX + RACC + FOREST + BOAR	4.5	0.007	43
FOX + BADG + RACC + FOREST	4.7	0.007	44
BADG + RACC + FOREST + WATER	4.8	0.006	45
FOX + BADG + RACC + WATER	4.9	0.006	46
RACC + FOREST + WATER + BOAR	4.9	0.006	47
FOX + BADG + RACC + SETT + FOREST	5.1	0.005	48
BADG + RACC + SETT + FOREST + WATER	5.1	0.005	49
FOX + BADG + RACC + SETT + WATER	5.2	0.005	50
FOX + RACC + FOREST + WATER	5.3	0.005	51
FOX + RACC + SETT + FOREST + WATER	5.8	0.004	52
FOX + BADG + RACC + SETT + FOREST + BOAR	5.9	0.004	53
FOX + BADG + RACC + SETT + WATER + BOAR	5.9	0.004	54
BADG + RACC + SETT + FOREST + WATER + BOAR	5.9	0.004	55
FOX + RACC + SETT + FOREST + WATER + BOAR	6.2	0.003	56
…			
null model	16.8	0.000	126
